# The New General Biological Property of Stem-like Tumor Cells (Part II: Surface Molecules, Which Belongs to Distinctive Groups with Particular Functions, Form a Unique Pattern Characteristic of a Certain Type of Tumor Stem-like Cells)

**DOI:** 10.3390/ijms232415800

**Published:** 2022-12-13

**Authors:** Daria D. Petrova, Evgeniya V. Dolgova, Anastasia S. Proskurina, Genrikh S. Ritter, Vera S. Ruzanova, Yaroslav R. Efremov, Ekaterina A. Potter, Svetlana S. Kirikovich, Evgeniy V. Levites, Oleg S. Taranov, Alexandr A. Ostanin, Elena R. Chernykh, Nikolay A. Kolchanov, Sergey S. Bogachev

**Affiliations:** 1 Institute of Cytology and Genetics of the Siberian Branch of the Russian Academy of Sciences, 630090 Novosibirsk, Russia; 2 Department of Natural Sciences, Novosibirsk National Research State University, 630090 Novosibirsk, Russia; 3 State Research Center of Virology and Biotechnology “Vector”, 630559 Novosibirsk, Russia; 4 Research Institute of Fundamental and Clinical Immunology, 630099 Novosibirsk, Russia

**Keywords:** internalization of double-stranded DNA, surface molecules of tumor stem cells, proteoglycans/glycoproteins, scavenger receptors, glycosylphosphatidylinositol-anchored proteins, Krebs-2 carcinoma, Epstein–Barr virus-induced B-cell lymphoma, RNA-seq

## Abstract

An ability of poorly differentiated cells of different genesis, including tumor stem-like cells (TSCs), to internalize extracellular double-stranded DNA (dsDNA) fragments was revealed in our studies. Using the models of Krebs-2 murine ascites carcinoma and EBV-induced human B-cell lymphoma culture, we demonstrated that dsDNA internalization into the cell consists of several mechanistically distinct phases. The primary contact with cell membrane factors is determined by electrostatic interactions. Firm contacts with cell envelope proteins are then formed, followed by internalization into the cell of the complex formed between the factor and the dsDNA probe bound to it. The key binding sites were found to be the heparin-binding domains, which are constituents of various cell surface proteins of TSCs—either the C1q domain, the collagen-binding domain, or domains of positively charged amino acids. These results imply that the interaction between extracellular dsDNA fragments and the cell, as well as their internalization, took place with the involvement of glycocalyx components (proteoglycans/glycoproteins (PGs/GPs) and glycosylphosphatidylinositol-anchored proteins (GPI-APs)) and the system of scavenger receptors (SRs), which are characteristic of TSCs and form functional clusters of cell surface proteins in TSCs. The key provisions of the concept characterizing the principle of organization of the “group-specific” cell surface factors of TSCs of various geneses were formulated. These factors belong to three protein clusters: GPs/PGs, GIP-APs, and SRs. For TSCs of different tumors, these clusters were found to be represented by different members with homotypic functions corresponding to the general function of the cluster to which they belong.

## 1. Introduction

### Historical Background and the General Logic of the Study

During our multiyear research, we revealed and characterized a novel general biological phenomenon, i.e., the ability of poorly differentiated cells of different geneses to internalize fragments of extracellular double-stranded DNA (dsDNA) into the cell [1,2]. Terminally differentiated cells belonging to different cellular communities do not internalize dsDNA as linear fragments or plasmids [2]. Following uptake into the cellular compartments, linear dsDNA fragments are processed, form a ring [3], and can remain in the cell in the form of episomes for up to 14 days [4].

The TAMRA-labeled dsDNA probe (a PCR fragment of cloned human *AluI* repeat) was designed and is consistently used in experimental practice [2].

**Figure 1:**
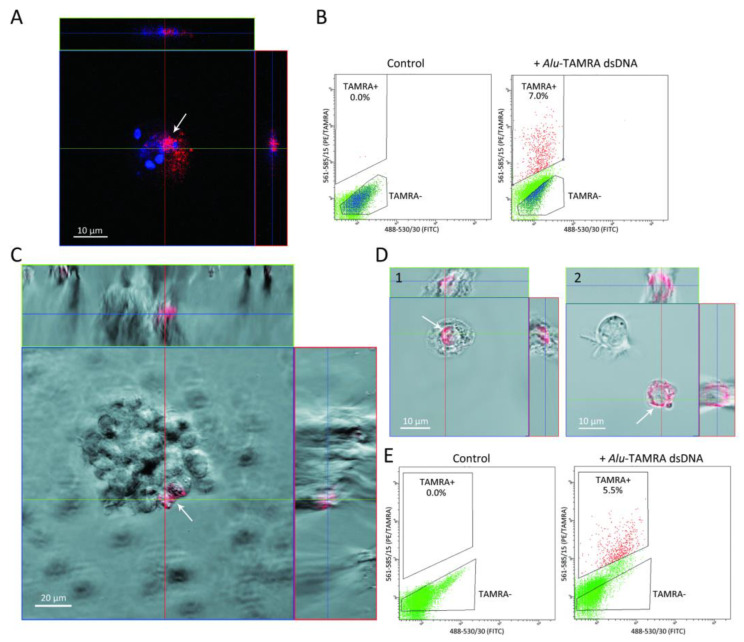
*Alu*-TAMRA dsDNA internalization into murine carcinoma Krebs-2 cells and human EBV-Induced B-cell lymphoma (HH47). (**A**) Orthogonal projections of Krebs-2 cells after incubation with the TAMRA dsDNA probe. (**B**) FACS assay of intact Krebs-2 cells (left panel) and those incubated with the *Alu*-TAMRA dsDNA probe (right panel). Orthogonal projections of the HH47 sphere (**C**) and individual cells (**D**) were produced using an LSM 780 NLO laser scanning microscope (Zeiss). The TAMRA signal corresponds to exogenous *Alu*-dsDNA residing inside the nucleus (1) or in the cytoplasm (2) of the cell. The X and Y views are shown on the right and top parts of the images, and the Z-plane projections are shown in the center. (**E**) FACS assay of the intact HH47 cells (left panel) and those incubated with the *Alu*-TAMRA dsDNA probe (right panel). All imaging procedures are described in detail in ref. [2]. The TAMRA signal (red) is shown by arrows.

Direct tumor-graft experiments for Krebs-2 carcinoma have shown that cells internalizing the TAMRA-labeled dsDNA probe (referred to as TAMRA+ cells in the text) (Figure 1A,B) are tumor stem-like cells (TSCs), yielding a stable, viable graft. RNA-seq analysis of TAMRA+ Krebs-2 cells identified a pattern of expressed genes that also characterize these cells as TSCs [5].

TAMRA+ cells of Epstein–Barr virus (EBV) induced B-cell lymphoma (Figure 1C–E) also have all signs of TSCs. It has been shown that they are clonogenic cells and constitute the sphere-organizing center. By secreting a set of specific chemokines, TAMRA+ cells provide conditions for the long-term existence of spherical aggregates [6]. B-cell lymphoma xenografts do not survive in the absence of TAMRA+ cells [7]. An analysis of RNA-seq reads demonstrated that TSC-specific genetic platforms are activated in TAMRA+ cells of EBV+ B-cell lymphoma ([1], see Table S1).

The revealed marker of TSCs, along with the eventual possibility of identifying the physical source of tumorigenic potential of the tumor graft, was used to develop and experimentally test the Karanahan technology, which has already been brought to the phase of pilot clinical trials [8]. The leading idea of this novel anticancer technology was to make it possible to eradicate TAMRA+ cells (TSCs) from the tumor focus in vivo. Devoid of its tumorigenic source, the tumor was eliminated by the body’s defense system. Complete eradiation of these cells from the tumor focus was always accompanied by healing of experimental mice (Figure S1). Using this approach, animals with tumor grafts of several incurable, rapidly progressive cancers were completely healed in numerous experiments [9,10,11].

The findings on tumor grafting, sphere formation, analysis of mRNA expressed in RNA-seq experiments, and survival of tumor-bearing mice after in vivo eradication of TAMRA+ cells from the tumor focus directly indicate that TAMRA+ cells of all the model tumors used in this study (murine Krebs-2, Lewis, hepatoma G-1, hepatocarcinoma G-29, RLS, Erlich and A-20 cancers; human B-cell lymphoma, and U87 glioblastoma) are TSCs [1,5,9,10,11,12,13].

The main question being asked over many years concerns the mechanism of internalization of dsDNA fragments into stem cells. In one of our previous studies [14], it was revealed that the heparin-binding site is critical for the internalization event.

To further develop this finding, we conducted a large experimental study aiming to characterize this phenomenon, which proved the validity of the initial observation [1]. Two model cell systems were selected: murine Krebs-2 ascites carcinoma as a model of in vivo cancer and the model of EBV-induced human B-cell lymphoma (HH47 [7]) spontaneously forming stable spherical aggregates as an in vitro transplantable culture [7].

The key binding sites were found to be heparin-binding domains within different cell surface proteins of TSCs—either the C1q domain, the collagen-binding domain, or domains of positively charged amino acids [1].

The central topic of the second part (the present article) of this comprehensive study revealed the fact that dsDNA binds to the aforementioned domains residing on TSC envelope factors and is internalized into the cellular compartments of this type of cells. An analysis of the experimental and analytical literature demonstrated that this trait (the presence of the heparin-binding domain) is typical of many cell surface factors belonging to proteins of the well-developed glycocalyx of three functional clusters: proteoglycans/glycoproteins (PGs/GPs), glycosylphosphatidylinositol-anchored proteins (GIP-Aps), and scavenger receptors (SRs). In other words, the interaction of the extracellular dsDNA fragments and the cell with their internalization occurs due to glycocalyx components (PGs/GPs, and GPI-APs) and the system of SRs, which are a distinctive feature of TSCs [1].

It became clear during this study that the interaction between dsDNA and TSC is rather complex and consists of several mechanistically distinct phases. The primary contact with cell membrane factors is mediated by electrostatic interactions. Firm contacts with cell membrane proteins then take place, followed by internalization of the complex formed between the factor and the dsDNA probe bound to it.

It was revealed in Part 1 of ref. [1] that the uptake of dsDNA fragments by the cell is determined by internalization of caveolae/clathrin vesicles and proteins bound to them. In the first instance, these are various receptors forming caveolae/clathrin signalosomes and specific lipid microdomains of the cell membrane [15] to which PGs/GPs, GPI-APs, and SRs belong.

PGs/GPs are an integral and indispensable part of internalization. Our experiments conducted in the first part of this study demonstrate that binding of dsDNA fragments to these glycocalyx proteins results in the formation of high local concentrations of bound ligands/polyanions. Nonetheless, the results of the experiments demonstrate that the interaction with PGs/GPs alone is not sufficient for an internalization event to occur. It is assumed that these proteins can contact receptors anchored in caveolae rafts or the cargo-clathrin substrate, thus providing transit of dsDNA molecules from the PG/GP-dsDNA complex to the internalization factor [16,17]. Therefore, PG/GP and the SR/GPI-AP/caveola or clathrin vesicle complex are the fundamental factors determining the interaction and internalization of dsDNA into cellular compartments, which were classified as group-specific cell surface factors of cancer stem-like cells responsible for their tumorigenic properties [1].

The aim of this study was to prove that the internalization factors containing the heparin-binding domain in their structure form three independent functional clusters of TSC surface proteins. For TSCs of different tumors, these clusters are represented by different members with homotypic functions corresponding to the general function of the cluster.

## 2. Results

### 2.1. Searching for the Putative Cell Surface Proteins Determining the Interaction of the TAMRA-Labeled dsDNA Probe with Krebs-2 and HH47 Tumor Stem-like Cells

#### 2.1.1. Search-Based Analysis of the Bioinformatic and Published Data on Functions of Putative Surface Proteins That Are Present on Krebs-2 and HH47 Cells and Are Responsible for Binding and Internalizing the dsDNA Probe

The data reported in the first part of this study [1] indicate the following. In the case of Krebs-2 cells, dsDNA bound to the cell surface proteins can be both internalized into cells and retained on their surface. For binding to the cell surface proteins, it is essential that positively charged domains (the collagen-like domain of SR-A and clusters of positively charged amino acids without apparent homology to the SR-A collagen-like domain) or a specific dsDNA-binding site (Cq1-like domain) are present in their structure. Internalization of dsDNA is abrogated after the disruption of lipid rafts with nystatin or blocking actin polymerization with cytochalasin D, indicating a possible association between dsDNA-binding proteins and lipid rafts, as well as the involvement of caveolae and micropinosomes in this process. Experiments with dextran sulfate 500,000 revealed that SRs may play a role in the import of dsDNA into the cell [18]. Therefore, the range of proteins responsible for the binding and internalizing of the dsDNA probe by Krebs-2 cells has been defined, i.e., proteins carrying a positively charged domain (the collagen-like domain of the SR-A or cluster of positively charged amino acids without apparent homology to the SR-A collagen-like domain) or a specific dsDNA-binding site (Cq1-like domain) in their structure and anchored to the plasma membrane. The indicated domains can reside both in the regions susceptible to proteinase K and in those unreachable for this enzyme (e.g., the forming caveola or macropinosome). Finally, the internalization factors appear to reside on the cytoplasmic membrane within the sites of the formation of caveolae with lipid rafts or macropinosomes, which mediate the rapid uptake of the dsDNA probe.

For HH47, the interaction between dsDNA and cells turned out to be somewhat different. The general mechanics of the interaction matches that in the Krebs-2 model and is associated with the presence of the SR-A collagen-like domain, a cluster(s) of positively charged amino acids, or the Cq1-like domain, which has overlapping heparin- and dsDNA-binding sites. According to the experiments with inhibitors, dsDNA internalization suggests the involvement of a clathrin-mediated mechanism (affected by chlorpromazine), caveolae-dependent endocytosis (affected by MβCD), and macropinocytosis (affected by cytochalasin D). Vesicle traffic is also active, as complete internalization occurs within 30–60 min, similar to the case of Krebs-2.

According to the obtained data, a bioinformatic analysis was conducted in combination with analysis of published data regarding the functions of proteins meeting the above-stated prerequisites, i.e., the proteins need to be plasma membrane-anchored proteins with a positively charged domain, which competitively binds heparin and dsDNA, or a consensus dsDNA-binding amino acid sequence homologous to the C1q domain of the complement (Table 1).

Thorough analysis performed in [19,20,21,22] indicates that the proteins analyzed in these studies did not carry a consensus sequence for the binding of heparin. The same applies to clusters of positively charged amino acids (arginine and lysine). A wide variety of very different arrangements of these amino acids with respect to each other gives rise to a positively charged region within the protein structure [23,24].

In this regard, we defined three domains binding heparin/dsDNA under experimental conditions and rely on them in our discussions. However, it should be noted that polyanion-binding sites within the structure of PGs, SRs, and GPI-APs are not limited to the sequences listed in Table 1.

These proteins can either be capable of drifting freely across the membrane, residing exclusively on the outer side of the cell, or can be incorporated into the structure of lipid rafts and thus internalized during caveolae/clathrin-dependent endocytosis or macropinocytosis. In other words, an integral “portrait” of the cell surface factors has been defined. The performed analysis indicates that proteins meeting the established prerequisites belong to three functional groups of the cell surface factors, namely proteoglycans/glycoproteins, SRs, and GPI-APs. C1q-like domains and clusters of positively charged amino acids are characteristic of proteoglycans, whereas collagen-like domains, which form a positively charged groove, or domains with clusters of positively charged amino acids are characteristic of SRs. GPI-raft-associated receptors may carry any of the aforementioned domains.

**Table 1:** Amino acid sequences of three specific polyanion/heparin/dsDNA-binding sites used as a reference for database searches.

Functional Group (Binding Site)	Amino Acid Sequence	Brief Description	Reference
SRs (collagen-like domain)	GRGNPGAPGKPGRSGSPGPKGQKGEKGSV	Heparin-binding sites are shown in blue	[25]
C1q protein (C1q-like domain)	VFTVTRQTHQPPAONSLIRFNAVLTNPQGDYDTSTGKFTCKVPGLYYFVYHASHTANLCVLLYRSGVKVVTFCGHTSKTNQVNSGGVLLRLQVGEEVWLAV	Heparin-binding sites are shown in blue; the DNA-binding site is shown in red	[26]
PGs/GPs and GPI-APs (cluster of positively charged amino acids)	IFLLVTLVTVCACWKPSKRKQKKLEKQNSLEYMDQNDD	The cluster of positively charged amino acids is shown in green	[20]

According to the performed analysis, the integral surface molecular profile of stem-like tumor cells is represented by the genes listed in Table 2.

An analysis of the obtained data allowed us to formulate the principle of organization of the stem-like tumor (and possibly some other poorly differentiated) cell surface factors as a concept of “group-specific cell surface factors, which form the profile of tumor stem-like cells”. Three functional groups were defined: the components of glycocalyx represented by PGs/GPs, SRs, and GPI-APs. In order to retain the functionality of each of these three groups, their member molecules must be present on the surface of tumor stem-like cells in sufficient amounts. These groups can be represented by different members, which simultaneously ensure both the specific functionality of the groups and form a unique pattern characteristic of tumor stem-like cells of a certain type. The essential property of all the factors included in the list is that their structures contain a polyanion (heparin/dsDNA/dextran sulfate)-binding domain represented by either a collagen-like domain, which forms a positively charged groove and is present in class A of SRs, a cluster(s) of positively charged amino acids, or a consensus site, which specifically binds heparin or dsDNA (but not RNA) (Table 1).

The transcriptomes of TAMRA+ Krebs-2 and HH47 cells were analyzed to prove the proposed concept. According to the concept, different molecules may be present on the membrane of Krebs-2 and HH47 stem-like cells, which, nevertheless, exhibit the same group-specific properties characteristic of the aforementioned distinct functional groups of the cell surface proteins. The intensity of dsDNA probe binding and internalization suggested the presence of a significant moiety of protein molecules responsible for the interaction between cells and dsDNA, which, in turn, presumes the active transcription of the respective genes.

#### 2.1.2. Identification of Group-Specific Genes for PGs, SRs, and GPI-APs Overexpressed in TAMRA+ Krebs-2 and HH47 Cells

The results of primary in silico transcriptome analysis and the consequently postulated existence of three functional groups of proteins overexpressed in TAMRA+ cells of murine Krebs-2 carcinoma and human EBV+ B-cell lymphoma were confirmed by validating qPCR targeting of the corresponding genes.

To assess the expression level of the genes for the defined group-specific factors, an RNA sequencing procedure was performed for TAMRA+ and TAMRA− cells of both cell models. The analysis revealed the overexpression of the genes characteristic of tumor stem-like cells in TAMRA+ cells of both Krebs-2 carcinoma [5] and HH47 [1]. Additionally, the analysis indicated that the overexpressed genes met the defined prerequisites, namely anchoring to the plasma membrane and the presence of heparin/dsDNA-binding sites. A comparative analysis was carried out for the genes from the three lists above: the genes of the generalized profile of tumor stem-like cells obtained during the published data search regarding the functional properties of the genes overexpressed in Krebs-2 and HH47 (Table 2).

The performed analysis allowed us to draw the following conclusions. TAMRA+ Krebs-2 and HH47 cells express the genes of the defined three groups at a high level. For both cell models, the lists of determined genes included both the genes identified during the “functional search” and additional genes (specific to each type of cancer stem cell) with all the defined group-specific prerequisites (Table 2). The overexpressed status of the genes encoding the soluble factors with polyanion-(heparin/dsDNA) binding domains was shown both in Krebs-2 (*Comp* and *Tnn*) and in HH47 (*Col15a1*, *Col22a1*, *Col5a2*, *Col6a1*, and *Col6a6*).

### 2.2. Quantitative Comparison of the Expression Levels of Several Group-Specific Factors by Real-Time PCR

**Figure 2:**
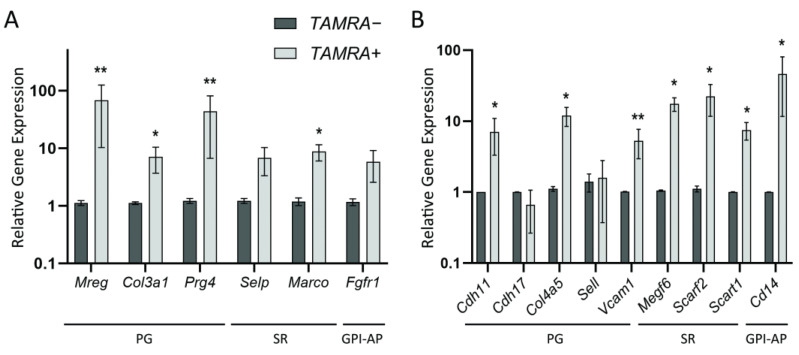
Assessing the relative expression levels for the genes of group-specific factors identified during the transcriptome analysis and presumably involved in dsDNA internalization. (**A**) Real-time PCR with cDNA obtained from Krebs-2 carcinoma. (**B**) Real-time PCR with cDNA obtained from HH47. For each gene, three to five independent PCR rounds were performed in triplicate. Means ± SEM are given, * *p* < 0.05, ** *p* < 0.01 (according to the Mann–Whitney U test).

A quantitative real-time PCR of the reverse-transcribed mRNAs of selected genes in both models was performed to validate the results of RNA sequencing and confirm the hypothesized concept. The obtained data confirmed the strong overexpression of *Mreg*, *Prg4*, *Col3a1*, *Selp*, *Marco*, and *Fgfr1* genes in Krebs-2 carcinoma, as well as *Cdh11*, *Col4a5*, *Vcam1*, *Megf6*, *Scarf2*, *Scart*1, and *Cd14* genes in HH47 (Figure 2). Such overexpression was not confirmed in the case of Cdh17 and Sell proteoglycans, which were expected to be overexpressed in TAMRA+ cells of HH47 according to transcriptome analysis. In general, the expression pattern indicates that the presumed concept was correct.

The present study is the first step towards understanding the addressed processes via bioinformatic analysis confirmed experimentally by qPCR validation of the transcriptome analysis based on an exploratory (single-round) RNA-seq. To obtain a reliable view of the expression of the analyzed factors, six independently obtained samples (sorted out TAMRA+ and TAMRA− cells) were used to validate the RT-qPCR procedures.

## 3. Discussion

In the Discussion section, we address the fundamental questions that have arose when analyzing the combined results of the first and the second parts of the study and rely on the same logic. This integral discussion is needed owing to the complex nature of relationships between the analyzed factors and functional clusters described in both parts of the study. The unique property of TSCs (that we call “functional borrowing”), which combines the functions of cells of other cellular systems by gathering specialized molecular patterns on their surface, is another important reason for leading a discussion in this form.

The findings allowed us to formulate a novel concept regarding one of the unknown principles of molecular organization of the “stem cell”, which determines its surface molecular profile and consists of two constituent elements.

The first element of the principle refers to the essential role played by three groups of factors, each with a particular function and need to be present on the surface of tumor stem-like cells.

There are several groups of protein factors (three described above) on the surface of tumor stem-like cells, each with particular functions. All three groups are represented on the surface of tumor stem-like cells as quantitatively dominating molecular patterns formed by transmembrane or membrane-anchored members of the PG/GP, SR, and GPI-AP families, which share one feature, namely a domain of amino acids with a definitely positive charge or a specific site for dsDNA binding. These three features (abundance, membrane anchorage, and the presence of a positive AA domain in the structure) determine two attributes of these cells, which distinguish them from their committed progeny, namely a general positive charge and the ability to bind and internalize polyanions, including heparin and dsDNA.

The second element of the principle of molecular organization of the surface proteins of tumor stem-like cells refers to the variability of particular members of three defined functional groups, which results in a molecular profile that is unique to the surface of particular tumor stem-like cells (originating both from different and, probably, from the same tumor), in addition to ensuring the prerequisite functionality.

Protein members of all three groups are obliged to be present on the membrane of any tumor stem-like cell to ensure the prerequisite functionality. However, these protein members can be different members of each group, resulting in a surface molecular profile that is unique to each particular type of tumor stem-like cell while retaining the general functionality specific to each group.

The findings obtained in this study and the novel view of the molecular features of the organization of surface proteins of tumor stem-like cells have aroused the two most intriguing questions, which, once answered, could significantly expand our understanding of tumor stem-like cell biology. These are:

- What can provide the general positive charge of tumor stem-like cells? and
- What inherent functions of tumor stem-like cells are ensured by such an exceedingly plastic mechanism?

### 3.1. What Can Provide the General Positive Charge of Tumor Stem-like Cells?

The performed analysis of published data resulted in some conclusions regarding the general positive charge found in Krebs-2 and HH47 tumor stem-like cells and probably characteristic of any poorly differentiated cells. As indicated in the first part of this study [1], the current paradigm does not presume the general positive charge of cells. Moreover, cells of a variety of tumors have been shown to be negatively charged [32,33,34]. Nevertheless, studies describing the molecular and biochemical features of the members of the defined functional groups have been published, which could prove and explain the positive charge formed on the surface of tumor stem-like cells. As previously mentioned, the surface of tumor stem-like cells carries proteins belonging to three functional groups, i.e., PGs, SRs, and GPI-APs, which are incorporated in its structure. According to the logic of the study, the positive charge of the cell surface is supposed to be ensured by the positively charged domains comprising arginine and lysine clusters within the collagen-like domain, clusters of positively charged amino acids, or consensus sequences present in the molecules of members of the aforementioned groups. Moreover, the farther from the cell membrane the maximum charge density is, the better. In this regard, the question remains as to how such a positive charge be formed in the presence of negatively charged molecules of core proteoglycans modified with covalently linked sulfated glycosaminoglycans.

In our opinion, there are several possible explanations for the prevalence of the general positive surface charge of TAMRA+ tumor stem-like cells. As reported in the first part of this study, the dsDNA probe is bound to the sites that also bind heparin. The analysis was performed using a vast body of accumulated information regarding the role of heparin as a guide interacting with and, thus, indicating the sites (molecules, domains, or AA clusters) on the cell surface, which can also bind dsDNA fragments. These are the aforementioned collagen-like domain(s), clusters of positively charged amino acids, and dsDNA-binding C1q-like consensus sequences (Table 1), and their spatial surface localization may ensure the formation of a positive charge on/around the cell surface. Here, a question arises about the negative charge formed by sulfated glycosaminoglycans (GAGs), which is commonly accepted to be prevalent. We suggest the following explanations for the migration of positively charged TAMRA+ cells towards the negative pole during electrophoretic separation and subsequent staining with TAMRA dsDNA.

Data regarding the variability of the PG/GP sulfation degree in tumor cells have been reported in recent studies [19,35,36]. A probable undersulfation of PGs/GPs in TAMRA+ cells could be the cause of the prevalence of a positive charge formed by the specific heparin/DNA-binding domains. In the case of PGs clustering, the positive charge is additionally strengthened by the spatial convergence of molecules carrying positively charged N-terminal domains [19,37,38,39]. In this case, there is a possibility of lateral (horizontal) multipoint interaction between linear dsDNA molecules and the “cap” of positively charged domains of clustered PGs/GPs. Such a mechanism has been described for the initial stages of formation of the extracellular matrix (ECM) with the involvement of collagen, heparin sulfate, fibronectin/integrins, hyaluronic acid, and multivalents of amyloid [19,38,40,41].

Collagen is known to have a domain for binding polyanions (heparin/dsDNA). Transcriptomic analysis revealed that both transcriptomes contain transcripts of the genes whose protein products (collagens) are secreted into the extracellular environment, where they form the ECM. Another overexpressed gene is integrin alpha, a collagen receptor present in both transcriptomes. Co-expressed in the same cell, this functional pair could mediate an indirect mechanism to bind extracellular dsDNA and surface proteins through the layer of ECM collagen(s) and its negatively charged specific receptor(s) [42].

Clusters of positively charged amino acids, as well as sites of specific binding of polyanions, are exposed outwards from the cell membrane and localized either at the N-termini of GP/PGs, spatially isolated from GAGs, which are fixed in the middle part of core proteins or in close vicinity of the membrane [19]. In this case, we can consider the possibility of local interaction between dsDNA and different parts of PG molecules located both at the N-termini and in the close vicinity of the membrane. If so, the 500 bp (~150 kDa) dsDNA fragment bypasses the negative charge of GAGs by forming a flexure above it. An analysis of amino acid sequences of proteoglycans overexpressed in Krebs-2 and HH47 revealed that polyanion-binding domains can be located both in the part most distant from the membrane of the molecule and in the direct proximity to it. It is possible that this part of the proteoglycan molecule(s) is responsible for binding dsDNA after proteolytic degradation of the glycocalyx coating [1].

Another intriguing phenomenon is the simultaneous intermolecular interaction between the domains of PGs and SRs or PGs and a specific receptor, as shown for the virion of herpes simplex virus or Ang-3 [19,36]. In this case, the observed pronounced multipoint interaction between the labeled dsDNA probe and the surface GPs/PGs of TAMRA+ cells (accumulation of a high density of dsDNA fragments in the perimembranous space) could ensure its effective internalization. In the first experimental part of this study [1], we demonstrated that cells exposed to proteolytic enzymes retain their capability of internalizing dsDNA fragments, whereas the detectable overall binding of these fragments to the cell surface subsides drastically, indicating the degradation of protein factors, which ensure the long-term retention of polyanions. Such a situation is possible in the presence of well-developed glycocalyx or sites of clustered GPs/PGs. In this case, the sulfated network of GAGs does not allow the enzyme to reach the membrane-adjacent polyanion-binding sites responsible for the internalization process [37].

The negative charge of GAGs could be compensated for by the interaction with the molecules of a variety of ligands binding to GAGs to initiate different signaling pathways [19,36,43]. Thus, albumin, with a weak positive charge of the side molecule chain, was shown to form a strong bond with GAGs [18,44,45,46].

Recent studies reported, for the first time, data regarding the possibility that the bulk of cells of the organism have a neutral or weakly positive total charge (tumor-reactive neutrophils) [32,45], whereas all tumor cells are charged negatively. This means a positively charged cell surface is theoretically allowed for stem-like tumor cells, providing a simple and universal mechanism for initial cell-to-cell contact, as well as for protection against reactive neutrophils.

### 3.2. What Inherent Functions of Tumor Stem-like Cells Are Ensured by Such an Exceedingly Plastic Mechanism?

To answer this question, we analyzed data on specialized cell communities whose members are characterized by the previously determined molecular patterns (PGs/GPs, SRs, and GPI-APs), constituting a major fraction of the structure of their surface and ensuring their functionality.

#### 3.2.1. Proteoglycans/Glycoproteins

Proteoglycans/glycoproteins are the molecules characteristic of a well-developed glycocalyx. The most prominent representatives with of pronounced glycocalyx are vascular endothelial cells and neutrophils. Glycocalyx is a labile polyanionic extracytoplasmic cell structure formed by membrane-anchored PGs/GPs associated with the extracellular matrix network and a variety of membrane-bound receptors. As an integral structure, the glycocalyx is involved in the processes of dynamic changes in blood flow parameters (shear stress). Normally, the main functions of glycocalyx are to regulate vascular tonus and homeostasis, maintain the interstitial fluid balance, and control the interaction of blood cells and signaling compounds with the vascular wall. Moreover, the charged glycocalyx network is believed to act as a macromolecular strainer, which repulses negatively charged molecules, erythrocytes, and platelets [47,48,49].

PGs act as the main basement structures for other glycocalyx components. As extended protein molecules (core proteins), they have multiple sites for covalent linking of GAG chains [50,51,52]. Membrane-associated glycocalyx PGs are subdivided into syndecans and glypicans (proteins anchored with GPI anchors in the membrane). Other PGs (biglycan, mimecan, perlecan, versican, and decorin) are soluble and bound to the glycocalyx through their ionic structural groups [19,53,54,55]. GPs comprise adhesion molecules (selectins, integrins, and the superfamily of immunoglobulins), receptors of intercellular signaling, and components of fibrinolysis and coagulation. These proteins regulate a variety of cellular functions, such as differentiation and maturation of lymphocytes, homing of immune cell precursors, recirculation of mature lymphocytes, antigen recognition, co-stimulation of lymphocytes, effective activation or anergy, proliferation induction and sustenance, and cytokine secretion, as well as maintenance of vascular homeostasis, fibrinolysis, and blood coagulation. Both PGs and GPs carry oligosaccharide groups with terminal sialic acid residues in their structure, which create specific sites for the binding of a variety of soluble signaling molecules on their surface. Binding of ligands and enzymes to the endothelial glycocalyx facilitates cellular signaling and enzymatic modification [43,56,57].

Recent studies have indicated that the glycocalyx is an essential component of the cell surface of both tumor cells in general and tumor stem-like cells in particular [58]. The glycocalyx of tumor cells is abundantly glycosylated. Glycosylation comprises sialylation, fucosylation, O-glycan truncation, and N- and O-glycan branching. Glycocalyx proteoglycans and glycoproteins in tumor cells participate in almost all processes that determine the biological peculiarities of malignified cells, such as epithelial-to-mesenchymal transition (EMT), motility, and metastasis, including the adhesion of tumor cells to the vascular endothelium and extracellular matrix, accumulation of growth factors in the perimembranous space and transduction of signals into the cell, blocking of proapoptotic signals, etc. Tumor cells regulate the recruitment and activation of myeloid-derived suppressor cells (MDSCs) by activating the synthesis of heparin sulfate and thereby changing the thickness and rigidity of the glycocalyx. Tumor cells are sensitive to shear stress, which is caused by interstitial flow and affects the integrity of the glycocalyx. Reducing the fluid flow rate around the tumor increases the survival of tumor cells [35,36,40,58,59,60,61]. Thus, PGs/GPs in tumor stem-like cells participate in the activation of signaling cascades, which determine the main tumorigenic properties of these cells, such as the EMT, motility, and metastasis, evading apoptosis and anoikis, and form a functionally specialized cohort of membrane proteins.

This protein pattern can be considered the first feature of the molecular organization of the cell surface, which is primarily characteristic of endothelial cells and neutrophils and acquired by tumor stem-like cells to ensure their survival in liquid tissues of the organism (evading complement-mediated lysis), as well as cell-to-cell interaction and extravasation into the essential tissues of the organism.

#### 3.2.2. Scavenger Receptors

Scavenger receptors comprise a heterogeneous (in terms of molecular organization) but functionally consolidated cohort of surface receptors. The most pronounced expression of SRs is observed in Kupffer cells (macrophages of the liver sinus capillaries), other stromal macrophages, and endothelial cells, as well as in some epithelial, stromal, and parenchymal cells. The most pronounced common property of SRs is their participation in the uptake of metabolic and other cellular “waste”, namely modified low-density lipoproteins, glycosylated proteins, damaged/senescent/apoptotic cells, aberrant erythrocytes and platelets, and a wide variety of other endogenous ligands. Different SR types are deeply involved in the key immune processes, including the presentation of antigens to naïve and inflammatory T-lymphocytes; regular and alternative macrophage (M) and T-helper (Th) differentiation; and the maturation, migration, and implementation of functions of immune cells [62,63,64]. Under active division of cells and tumor cells in particular, SRs provide proliferating cells with nutrients (cholesterol, phospholipids, proteins, etc.) [65]. Moreover, the expression of SRs in tumor cells facilitates their proliferation, as well as the growth and vascularization of the tumor tissue per se [66,67,68,69,70]. The wide range of functional capabilities of SRs is determined by their diversity, multiligand affinity, and capability of forming intricate complexes with receptors of other types (e.g., TLRs or integrins). Participating in such complexes, SRs modulate the functions of partner receptors and thereby affect their signaling pathways [62]. Thus, the presence of such a multifunctional cohort of proteins as SRs on the surface of tumor stem-like cells ensures their active metabolism by providing an effective influx of nutrients and other essential metabolites. Some other discovered functional capabilities of SRs may contribute to mimicking immune cells and consequent evasion of immune surveillance by cancer stem cells.

The aforementioned facts imply that SRs expressed by tumor stem-like cells can be considered the second feature of the molecular organization of the cell surface, which is primarily characteristic of stromal macrophages acquired by tumor stem-like cells.

#### 3.2.3. Glycosylphosphatidylinositol-Anchored Proteins

Glycosylphosphatidylinositol-anchored proteins are a family of membrane-anchored proteins with a covalently bound glycosylphosphatidylinositol residue serving as an anchor. Approximately 150 GPI-APs have been discovered in humans. The main feature of these molecules is their association with membrane microdomains (cholesterol rafts) and participation in signalosome formation. The following main types of GPI-APs are known: enzymes, “navigation” receptors, adhesion molecules, receptors and coreceptors of growth factors, and receptors mediating the formation of immune complexes. A GPI anchor enables the apical targeting of proteins in polarized cells. Similar to SRs, GPI-APs participate in endocytosis of specific ligands, including nutrients (folic acid receptor, FR), via lipid rafts [30,31,71,72,73]. The expression of GPI-APs (including those with polyanion-binding domains) with different functions (adhesion molecules, growth factor receptors and coreceptors, and immune complexes), their polarization or hydrolytic cleavage, and active endocytosis via cholesterol rafts/caveolae may indicate active metabolic processes, which is typical of tumor stem-like cells.

This means that the presence of GPI-APs can be considered the third feature of the molecular organization of the cell surface, which is primarily characteristic of cells with an active metabolism acquired by tumor stem-like cells.

#### 3.2.4. Caveolae-Dependent Transport Is Typical of Cells with Intensive Metabolism

Both types of proteins, SRs and GPI-APs, function by being associated with cholesterol rafts, which, in turn, are essential components of caveolae. Caveolae and therefore rafts are the sites mediating the molecular organization of the aforementioned groups of proteins, SRs and GPI-APs. The balance between the members of these groups in rafts appears to depend on the type or physiological state of the cell and may vary accordingly. Caveolae are believed to serve as mechanosensors and regulators of lipid homeostasis. One of the main physiological functions of caveolae is their ability to act as a damper under stressful tension of the cell membrane [74,75]. Caveolae are abundant in cells of adipose tissue, muscles, and the endothelium, comprising up to 50% of the surface area. It is supposed that specific transmembrane receptors do not participate in the formation of caveolar domains [76,77,78,79,80,81]. Caveolae-mediated endocytosis contributes to the regulation of cell motility, the cell cycle, the polarity of cells, apoptosis, gene transcription, cell tropism, drug resistance, and overall homeostasis and metabolism [18,82]. Caveolae enable the deposition of cholesterol, sphingomyelin, and ceramides [83,84,85,86], the import of lipids and fatty acids into the cell [87,88], and endothelial transcytosis of large molecules [39,89]. Caveolae underlie the formation of signaling complexes of various proteins—SRs and GPI-APs in particular. Such participation of caveolae in a variety of cellular functions suggests that they are essential for active metabolic processes characteristic of tumor stem cells.

Thus, the presence of caveolae, as well as caveolae-mediated transport in TAMRA+ cells (which is circumstantially confirmed by the overexpression of cholesterol raft-associated GPI-APs and SRs, as well as by the abrogation of dsDNA internalization after exposure to compounds inhibiting the formation of rafts in Krebs-2 and HH47 cells) can be considered the third feature of the molecular organization of the cell surface, which is primarily characteristic of epithelial, fat, and muscle cells, where it ensures the response to plastic deformation, accumulation of fats and fatty acids, maintenance of lipid homeostasis, trafficking of large molecules, signal transduction, and other attributes of the active metabolism acquired by tumor stem-like cells.

**Figure 3:**
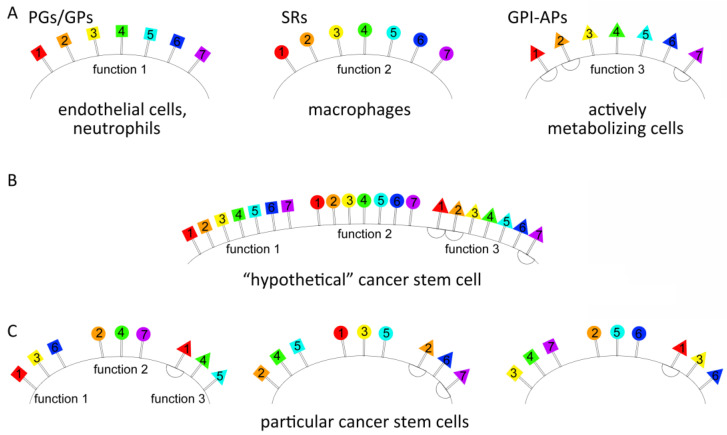
The principle of molecular organization of a “stem cell”, which determines its surface molecular profile and consists of two postulates. Molecular organization of surface proteins in tumor stem-like cells is based on the fact that three functional groups of proteins need to be present on their surface. These groups need to retain their functional properties, regardless of their internal composition, which may differ, thereby forming a unique surface profile in tumor stem-like cells from both multiple and single tumors. (**A**) Specialized cells with particular functions also exerted by cancer stem cells. The geometric figures and numbers correspond to specific protein groups providing an appropriate functionality characteristic of the indicated cell type. Squares—proteoglycans/glycoproteins (PGs/GPs); circles—scavenger receptors (SRs); triangles—glycosylphosphatidylinositol-anchored proteins (GPI-APs). (**B**) The molecular profile of the surface of a “hypothetical” cancer stem cell. (**C**) The molecular profiles of the surface of particular cancer stem cells.

The obtained data provide novel insight into the previously unknown biological peculiarities of tumor stem-like cells. Cancer stem cells have a particular surface profile associated with the overrepresentation of proteins with specific functions belonging to three major groups, namely PGs/GPs, SRs, and GPI-APs (Figure 3). Thus, the surface of tumor stem-like cells agglomerates three functional groups, which are normally characteristic of and limited to particular types of cells. Such a peculiarity allows different and unique capabilities normally characteristic of different specialized cells to be combined in a single stem-like tumor cell. Therefore, the positive charge on the surface of tumor stem-like cells is a biological property of such cells, which manifests as the initial/primary binding of TAMRA dsDNA fragments to the positively charged “coat”. Such an organization of the cell surface of tumor stem-like cells appears to allow them to evade tissue-specific rejection.

The main outcome of this study is the assumption that tumor stem-like cells from different tumors have no shared unique surface markers. Instead, they have shared functions vital to any tumor stem-like cell and assigned to several functional groups. These groups are agglomerated on the same tumor stem-like cell but can be composed of molecules differing not only between cancer stem cells of different origin but possibly even within a subpopulation of cancer stem cells originating from the same tumor. Each molecule belonging to a given group exerts exactly the same function as all other members of the group. As a result, in individual tumor stem-like cells, regardless of whether they originate from different or the same tumor, the appropriate processes (e.g., the internalization of extracellular material) occur with the participation of different members of the same “team”. In this case, the individual analysis of different types of stem-like tumor cells produces an apparent absence of shared specific factors, which could determine the function. Indeed, the function is implemented by different factors with the same properties, which determines the unconditional implementation of the same function. Such an assumption could be the rationale for the failure in searching for a “universal protein marker of tumor stem-like cells”.

## 4. Materials and Methods

### 4.1. Experimental Design and Study Logic

1. Results obtained in the first part of the study suggested the role played by proteins with a heparin-binding domain in binding and internalization of dsDNA fragments [1].

2. Bioinformatic analysis of factors carrying this domain made it possible to divide them into three functional groups. This was the quintessence of the birth of the postulate about the existence of three functional groups capable of binding extracellular dsDNA fragments.

3. To prove the postulate, two sets of RNA sequencing data obtained in the laboratory of the induced cell process IC&G SB RAS for murine Krebs-2 carcinoma and human EBV-induced B-cell lymphoma were analyzed, and the results confirmed the postulate.

4. Results of the transcriptome analysis were validated using standard qPCR. To obtain more reliable results, qPCRs were performed using specimens of TAMRA+/− cells obtained in six independent sorting procedures.

### 4.2. Tumor Model

The Epstein–Barr virus-induced human B-lymphoma cell line (EBV-induced B-lymphoma) derived from the aspirate of bone marrow cells of a patient diagnosed with multiple myeloma was obtained from the Research Institute of Fundamental and Clinical Immunology (Novosibirsk, Russia). This cell culture was designated as HH47 [7]. HH47 was stored in the Cell Line Repository of the Institute of Cytology and Genetics of the SB RAS (No. HSCC00081).

The cells were cultured in Dulbecco’s Modified Eagle Medium (DMEM, Gibco, Waltham, MA, USA) supplemented with 40 μg/mL gentamycin sulfate and 10% fetal bovine serum (FBS) (HyClone, Logan, UT, USA) at 37 °C and 5% CO_2_ in a CO_2_ incubator (Memmert, Eagle, WI, USA). To prepare the suspension, cells were gently detached from the plastic by pipetting and sedimented at 400 g for 5 min. The cell pellet was then resuspended in DMEM.

### 4.3. Isolation of HH47-Internalizing, TAMRA-Labeled dsDNA

*Alu*-TAMRA dsDNA is a 500 bp fragment of the human *Alu* repeat labeled with dUTP-5′-TAMRA (Biosan, Novosibirsk, Russia) by PCR [2]. HH47 cells were incubated with *Alu*-TAMRA dsDNA (0.5 µg per 2 × 10^6^ cells in 500 µL of DMEM medium (Gibco, NY, USA)) for 1 h at room temperature. The TAMRA+ and TAMRA− subpopulations were separated on a BD FACSAria III (BD, NJ, USA) cell sorter equipped with a 100 µm nozzle at an event rate varying between 2000 and 3500 cells. A 561 nm laser and a 585/15 channel (also known as a PE channel) were used to detect TAMRA fluorescence.

Several rounds of flow cytometry-based cell sorting were used to obtain sufficient amounts of TAMRA+ cells. The obtained fraction of cells (referred to as “TAMRA+ cells”) contained 80–90% TAMRA+ cells and 10–20% TAMRA− cells. The fraction of TAMRA− cells was pure.

After sorting, microscopic analysis of the percentage of TAMRA+ cells was performed using an AxioImager M1 laser scanning microscope (Carl Zeiss, Oberkochen, Germany) in the Collective Use Facility for Microscopic Analysis of Biological Objects, Siberian Branch of the Russian Academy of Sciences.

### 4.4. RNA Isolation and RNA-seq

Total RNA from HH47 TAMRA+ and TAMRA− cells was isolated using TRIzol Reagent (Thermo Fisher Scientific, Waltham, MA, USA) following the manufacturer’s instructions. Libraries were constructed using an Illumina TruSeq RNA sample preparation kit, v.2. (Illumina, CA, USA). The libraries were loaded on MiSeq (Illumina) using a MiSeq Reagent Kit v.3 (Illumina).

### 4.5. Transcriptome Analysis

The obtained sets of reads were cleared of (i) adapter sequences using the TrimGalore package (Babraham Institute, Babraham, UK), and (ii) short (fewer than 20 nucleotides) reads remained after trimming adapter sequences. The resulting reads were mapped to the target sequence using the ReadsMap package (Softberry Inc., Mount Kisco, NY, USA) in “solid” mode. The target sequence was obtained by linking the retrieved transcripts (*H. sapiens* mRNAs available for download as a KnownGeneMrna.txt file from the site of the Genomic Institute, University of California, Santa Cruz, CA, USA) into a single contig. In this contig, individual transcripts were separated from by inserting spacers, (N)_n_, consisting of masked nucleotides, where N is the masked nucleotide (cannot be recognized as a valid target for mapping) and n is the specified spacer length (determined by the window size during the RNA sequencing and used to avoid mismapping of paired reads to different transcripts).

Reads were mapped in the strict mode (no more than a single nucleotide mismatch/deletion/insertion per alignment allowed). Only the best variants for each pair of reads were kept from the resulting set of alignments.

The results of transcriptome analysis preformed previously for murine Krebs-2 carcinoma [5] were also used in this study.

### 4.6. Real-Time PCR and Primer Selection

HH47 and Krebs-2 ascites tumor cells were sorted into TAMRA+ and TAMRA− subpopulations, and RNA was isolated. CDNA was synthesized for each group using a Mint-2 cDNA synthesis kit (Evrogen, Moscow, Russia). PCR primers for coding regions of each gene were designed using Vector NTI v.9 (Life Technologies, Wilmington, DE, USA) software (Table S1). The primers were tested for specificity using the total cDNA as a template. Real-time qPCR was run on a CFX Connect real-time PCR detection system (BioRad Inc., Hercules, CA, USA) using BioMaster HS-qPCR Hi-Rox SYBR Blue (2×) reagent (Biolabmix, Novosibirsk, Russia). Murine *Actb* and human *Rplp0* genes were used as references.

The cycling parameters were as follows: 95 °C for 10 min, 40 cycles of 95 °C for 30 s, 60 °C for 30 s, 72 °C for 30 s, with a final melting step with slow heating from 6 °C to 95 °C. All reactions were run in triplicate within a single run, and negative control reactions without reverse transcription reaction and template were also performed. A total of six independent sorting procedures were performed, followed by RNA extraction and cDNA synthesis for real-time PCR for Krebs-2 cells, with four procedures performed for HH47.

### 4.7. Statistical Analysis

Real-time PCR data indicate the fold differences (ΔΔCt) in gene expression between the TAMRA+ and TAMRA− samples. Means ± the standard error of the mean (SEM) are shown in the diagrams (*n* = 3–6). The reliability of the results was assessed using the Mann–Whitney U test available in the Statistica package version 10 (StatSoft, Tulsa, OK, USA).

## 5. Conclusions

According to the obtained results, a concept of a novel approach to detecting stem cells of different origin, including tumor stem-like cells, using a dsDNA marker was formulated. The main property of such cells allowing for their marking and detection in the bulk of differentiated tumor cells is their overall positive charge and surface expression of certain proteins responsible for binding and internalizing dsDNA. In this case, the labeled dsDNA probe can be a suitable tool for detection of tumor stem-like cells both in vitro and in vivo.

## Figures and Tables

**Table 2:** General comparative table of the group-specific factors of the integral cell surface molecular profile of a “hypothetical” TSC, i.e., carrying all possible members of the three functional groups (PGs/GPs, GPI-APs, and SRs) and those of real Krebs-2 and HH47 TSCs.

Groups of the Membrane-Anchored Proteins with the Same Particular Function	“Hypothetical” Tumor Stem-like Cell Based on the Published Data [20,27,28,29,30,31]	Krebs-2	HH47
PGs/GPs	*Adipoq* *Caprin2* *Cdh13* *Clec4* *Col12A1* *Fcn2* *Gldn* *Gpc3* *Hip* *Itgam* *Ncam* *Otol1* *Sell* *Selplg*	*Apoc1* *Catsperg2* *Cd200* *Col3a1* *Col6a2* *Cldn1* *Dsc2* *Dnaja4* *Fcna* *Adgrg3* *Hepacam* *Itm2a* *Itga9* *Itln1* *Jsrp1* *Lyve1* *Mreg* *Nectin1* *Ncam* *Psd2* *Pgr* *Selplg* *Rab15* *Tnfrsf13c*	*Acvr1* *Cdh11* *Cdh17* *Chodl* *Col4a5* *Col4a6* *Col7a1* *Cthrc1* *Fcrl3* *Gpc* 1 *Gpc*2 *Igf1r* *Itga10* *Itga5* *Kcnip3* *Kel* *Ncam* *Sell* *Podxl* *Ptprm* *Tenm4* *Tspan15* *Treml4* *Vcam1*
SRs	*Marco* *Megf* *Scara* *Scarb*	*Marco*	*Megf6* *Scarf2* *Scart1*
GPI-APs	*Aamp* *Ache* *Car4* *Cd14* *Cd24a* *Cd48* *Cd55* *Cd59a* *Cdh1* *Ceacam5* *Cntfr* *Cpm* *Fgfr* *Folr1* *Gpc3* *Gpihbp1* *H2-Q7* *Ly6a* *Lypd1* *Mdp1* *Msln* *Nrp1* *Parp1* *Plaur* *Robo* *Thy1*	*Cd55* *Fgfr1*	*Cd14* *Gdpd5*

## Data Availability

The data that support the findings of this study are available from the corresponding author upon reasonable request.

## Supplementary Materials

The supporting information can be downloaded at: https://www.mdpi.com/article/10.3390/ijms232415800/s1. Reference [90] is cited in the supplementary materials.
