# Supplementary material for: The New General Biological Property of Stem-like Tumor Cells (Part II: Surface Molecules, Which Belongs to Distinctive Groups with Particular Functions, Form a Unique Pattern Characteristic of a Certain Type of Tumor Stem-like Cells)
Source: Int J Mol Sci. 2022 Dec 13;23(24):15800. doi: 10.3390/ijms232415800 (PMC9785054; doi:10.3390/ijms232415800)
Supplement: Supplementary file 1 [file ijms-23-15800-s001.zip › Supplementary Material S1.pdf]

Supplementary Material S1

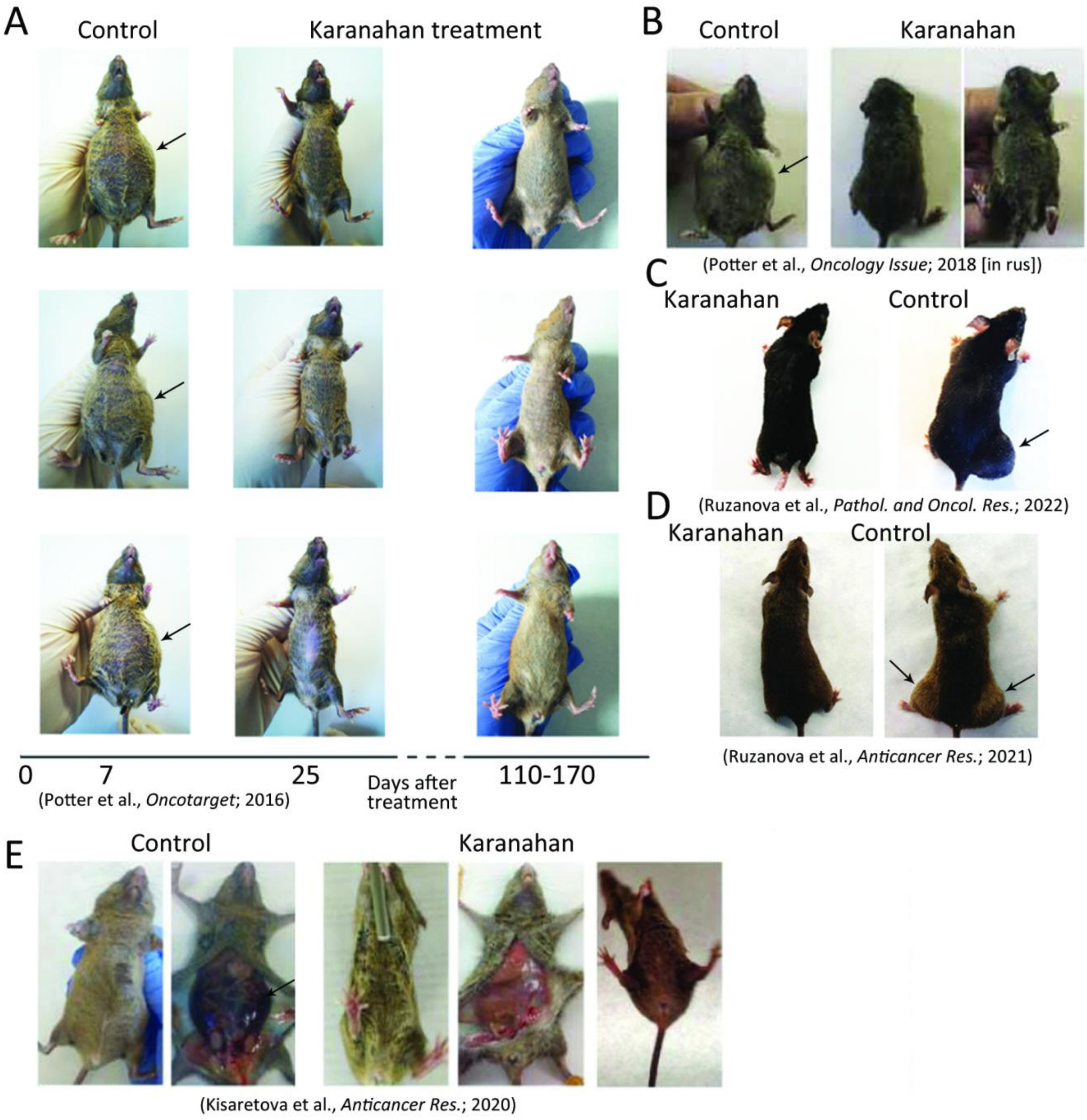

Figure S1. Efficacy of the Karanahan approach shown in different mouse tumor models. Overall visual condition of experimental animals prior and after the therapy is shown. (**A**) Krebs-2 ascites. Mice before the therapy (left panels), during ascites regression period (~day 25 post the first CP injection, middle panels) and cured mice on day 150 of the experiment (right panels) [11]. (**B**) Ascitic tumor G-29 [90]. (**C**) Lewis carcinoma with an intramuscular graft [10]. (**D**) Solid Krebs-2 tumor. Tumors were grafted intramuscularly in the two hind paws of the mouse [12]. (**E**) RLS tumor [9]. The presence of a tumor is indicated by an arrow.

Supplementary Material S1 references are presented in the main text of the manuscript.
