# Supplementary material for: The New General Biological Property of Stem-like Tumor Cells (Part II: Surface Molecules, Which Belongs to Distinctive Groups with Particular Functions, Form a Unique Pattern Characteristic of a Certain Type of Tumor Stem-like Cells)
Source: Int J Mol Sci. 2022 Dec 13;23(24):15800. doi: 10.3390/ijms232415800 (PMC9785054; doi:10.3390/ijms232415800)
Supplement: Supplementary file 1 [file ijms-23-15800-s001.zip › Supplementary Material S2.pdf]

Supplementary Material S2

Table S1. Primers used for real-time PCR.

Model	Target Gene Transcript ID	Forward Primer (Tm, ºC) Reverse Primer (Tm, ºC)	Product Size, b.p.
Krebs-2	*Mreg* ENSMUST00000048860.9	5′–AAGTGAGGAACCGATGGAGACGA–3′ (58.6) 5′–AGCATCTCCCGGGCTTTCCT–3′ (58.7)	130
	Col3a1 ENSMUST00000087883.13	5′–GGCCCTGGTGAGAGAGGTGAACAT–3′ (61.3) 5′–GGACCACCAGGACTACCACGTTCA–3′ (60.8)	221
	Prg4 ENSMUST00000164600.8	5′–GATGGACTGACTACGCTGCGCAA–3′ (61.1) 5′–GGTGAAGCGCCAGTACTGAGAATCC–3′ (61.1)	207
	Selp ENSMUST00000162746.2	5′–ACGACTCCTGGACAGGGCACAA–3′ (60.3) 5′–TTTGACCGCTCTGCACATGGG–3′ (60.4)	174
	Marco ENSMUST00000027639.8	5′–CCCGGGAATTGCTGGGAATC–3′ (59.9) 5′–GGGCTTCCAGTGTCTCCCTTTCT–3′ (58.7)	138
	Fgfr1 ENSMUST00000084027.13	5′–TGCCTGTGGAGGAACTTTTCAAGC–3′ (60.4) 5′–CAATGCGGTCCAGGTCTTCCAC–3′ (60.2)	162
	Actb ENSMUST00000100497.11	5′–GGTGTGATGGTGGGAATGGG–3′ (55.9) 5′–TCTCCATGTCGTCCCAGTTGG–3′ (56.3)	127
EBV+ B-lymphoma	*Cdh11* ENST00000268603.9	5′–ATCGCCATCCTCGCCTGCAT–3′ (61.2) 5′–GGGTGGCAATATCAAAGGCTTCTG–3′ (59.7)	181
	Cdh17 ENST00000027335.8	5′–CCCCTCAGTGCACCTGGAAGTCTCA–3′ (64.3) 5′–CCGACCCCCATCATTGATGC–3′ (59.4)	219
	Col4a5 ENST00000328300.11	5′–GCCAGGTACCCGTGGTTTGGAT–3′ (60.6) 5′–CAAGTCTTGACCGTGGGCTCTTTT–3′ (59.6)	217
	Sell ENST00000236147.6	5′–ACCATGGACTGTACTCACCCTTTGG–3′ (59.1) 5′–AATCTGGTGCTGATAGAGGCTCACA–3′ (58.1)	181
	Vcam1 ENST00000294728.7	5′–ATGTGAAGGAATTAACCAGGCTGGA– 3′ (58.9) 5′–CCGCTTTTTTCTTCAGGATTATCCA–3′ (58.1)	185
	Megf6 ENST00000356575.9	5′–GCTTCCACGGCCACTTCTGTGA–3′ (60.9) 5′–TGGCCCCTTCTGCAATCCAG–3′ (59.5)	172

	Scarf2<br>ENST00000622235.5	5′–CCAGCGACAAATCGGCGCATAC–3′ (63.1)<br>5′–AGCTCAGCGGCCAACATGGA–3′ (60.2)	220
	Scart1<br>ENST00000640237.1	5′–ACCGCCTGGGTGGACAACAT–3′ (59.0)<br>5′–CCTGTGGCCTGTCCTCTGACAATC–3′ (58.6)	129
	Cd14<br>ENST00000302014.11	5′–CCCACAGCCTAGACCTCAGCCACAA–3′ (64.1)<br>5′–CCTGTTCAGTCTGTTGCAGCTGAGA–3′ (59.6)	182
	Rplp0<br>ENST00000392514.9	5′–AGGCCTTCTTGGCTGATCCATCT–3′ (59)<br>5′–TATCCTCGTCCGACTCCTCCGA–3′ (59)	135
